# Toll-like receptor 2 activation primes and upregulates osteoclastogenesis via lox-1

**DOI:** 10.1186/s12944-018-0787-4

**Published:** 2018-06-02

**Authors:** Kimiko Ohgi, Hiroshi Kajiya, Kazuko Goto-T, Fujio Okamoto, Yasunori Yoshinaga, Koji Okabe, Ryuji Sakagami

**Affiliations:** 1 Department of Odontology, Fukuoka Dental College, Fukuoka, 8140193 Japan; 2 Department of Physiological Science and Molecular Biology, Fukuoka Dental College, Fukuoka, 8140193 Japan; 3 Department of Dental Hygiene, Fukuoka College of Health Sciences, Fukuoka, 8140193 Japan

**Keywords:** Dyslipidemia, Osteoclastogenesis, Toll-like receptor, Lectin-like oxidized low-density-lipoprotein receptor 1

## Abstract

**Background:**

Lectin-like oxidized low-density-lipoprotein receptor 1 (Lox-1) is the receptor for oxidized low-density lipoprotein (oxLDL), a mediator in dyslipidemia. Toll-like receptor (TLR)-2 and − 4 are receptors of lipopolysaccharide (LPS) from *Porphyromonas gingivalis*, a major pathogen of chronic periodontitis. Although some reports have demonstrated that periodontitis has an adverse effect on dyslipidemia, little is clear that the mechanism is explained the effects of dyslipidemia on osteoclastogenesis. We have hypothesized that osteoclast oxLDL has directly effect on osteoclasts (OCs), and therefore alveolar bone loss on periodontitis may be increased by dyslipidemia. The present study aimed to elucidate the effect of Lox-1 on osteoclastogenesis associated with TLRs in vitro.

**Methods:**

Mouse bone marrow cells (BMCs) were stimulated with macrophage colony-stimulating factor into bone marrow macrophages (BMMs). The cells were also stimulated with synthetic ligands for TLR2 (Pam3CSK4) or TLR4 (Lipid A), with or without receptor activator of nuclear factor kappa-B ligand (RANKL), and assessed for osteoclastogenesis by tartrate-resistant acid phosphatase (TRAP) staining, immunostaining, western blotting, flow activated cell sorting (FACS) analysis, real-time polymerase chain reaction (PCR), and reverse transcription PCR.

**Results:**

Lox-1 expression was significantly upregulated by Pam3CSK4 and Lipid A in BMCs (*p* < 0.05), but not in BMMs. FACS analysis identified that Pam3CSK4 upregulated RANK and Lox-1 expression in BMCs. TRAP-positive cells were not increased by stimulation with Pam3CSK4 alone, but were increased by stimulation with combination combined Pam3CSK and oxLDL. Expression of both Lox-1 and myeloid differentiation factor 88 (MyD88), an essential adaptor protein in the TLR signaling pathway, were suppressed by inhibitors of TLR2, TLR4 and mitogen-activated protein kinase (MAPK).

**Conclusions:**

This study supports that osteoclastogenesis is promoted under the coexistence of oxLDL by TLR2-induced upregulation of Lox-1 in BMCs. This indicates that periodontitis could worsen with progression of dyslipidemia.

## Background

Osteoclasts (OCs) are multinucleated, bone-adhering cells, formed by fusion of mononuclear monocyte/macrophage progenitors. OCs require macrophage colony-stimulating factor (M-CSF) for proliferation and receptor activator of nuclear factor kappa-B ligand (RANKL) for fusion and maturation [1–5]. OCs act by extruding acid onto the bone surface and dissolving inorganic components within the bone matrix [5]. Periodontitis is a chronic disease caused by bacterial infection in the gingiva [6, 7], resulting in resorption of alveolar bone. It is reported that lipopolysaccharide (LPS), a major constituent of Gram-negative bacteria, can stimulate OC formation and bone resorption through activation of Toll-like receptors (TLRs) [8–10]. *Porphyromonas gingivalis* (P. gingivalis) is an anaerobic Gram-negative oral bacterium involved in periodontitis. LPS derived from P. gingivalis is different to that derived from other bacteria and acts as an agonist of both TLR4 and TLR2 [11, 12]. P. gingivalis aggravates periodontal bone resorption through differential regulation of TLR2 and TLR4 signaling pathways in a RANKL-dependent manner [13]. Although TLR ligands have been shown to have stimulatory or inhibitory functions on osteoclastogenesis in vitro, TLR4 ligand has been shown to be a potent stimulator of bone loss in vivo [14]. It has been reported that P. gingivalis LPS stimulates periosteal OC formation due to induction of RANKL in osteoblasts by activation of TLR2 [15]. Of note, alveolar bone loss is induced by P. gingivalis through TLR2 in mice [13, 16, 17]. Conversely, synthetic TLR2 ligand inhibits OC formation in mouse bone marrow macrophages (BMMs) stimulated with macrophage colony-stimulating factor (M-CSF) and RANKL [18, 19]. Although RANKL induces differentiation of BMMs to OCs, simultaneous addition of LPS inhibits this process [19, 20]. However, LPS stimulation at late-stage osteoclastogenesis enhances the survival and activation of OCs [20–22]. Although *P. gingivalis* has known to promote osteoclastogenesis and bone resorption which types of TLR receptor, such as TLR2 and/or TLR4 promote bone resorption remains unclear.

Lectin-like oxidized low-density lipoprotein receptor-1 (Lox-1) was discovered as a receptor for oxidized low-density lipoprotein (oxLDL) in endothelium and vascular-rich organs [23]. Lox-1 is a multi-ligand receptor that recognizes many ligands, such as activated platelets [24], neutrophils [25], apoptotic/aged cells [26], and bacteria [27], and is expressed in many cell types [28–30]. Bone-resorbing OCs and bone-forming osteoblasts have been reported to express Lox-1, and Lox-1^−/−^ mice have decreased bone mass in the steady state but are resistant to inflammatory bone destruction because of the impairment of osteoblastic RANKL expression in response to inflammation [31].

The pathology of both periodontitis and dyslipidemia involves OCs, and these lifestyle-related diseases are exacerbated by stimulation with TLRs and Lox-1, respectively. Consequently, although these disorders are reported to be associated with each other, the mechanism for this is unclear [32–34]. Some reports have shown that periodontitis increases the risk of atherosclerosis in vivo and in vitro [35–38], and that periodontitis worsens in the apolipoprotein E (ApoE)−/− hyperlipidemia model [33, 35, 39]. However, there are few reports describing how dyslipidemia exacerbates periodontitis.

The purpose of this study was to clarify whether osteoclastogenesis is upregulated through Lox-1 by TLR stimulation at early- and late-stage. We showed that osteoclastogenesis was accelerated by activation of TLRs through upregulation of Lox-1 expression during bone marrow cell (BMC) differentiation into BMMs, suggesting dyslipidemia increases the risk of periodontitis.

## Methods

### Cell culture

All procedures were approved by the Council on Animal Care of Fukuoka Dental College (13013). Mouse BMCs were obtained from the tibia and femora of 4- to 5-week-old ddY male mice. All mice were euthanized by cervical dislocation under anesthesia by inhalation of isoflurane at 0.5–5% with oxygen. BMCs were cultured in α-Minimum Essential Medium (α-MEM; Invitrogen, Grand Island, NY, USA) containing 10% fetal bovine serum (FBS; Biowest Nuaille, France) and antibiotics (100 U/ml penicillin G and 0.15 mg/ml streptomycin sulfate). After overnight culture to differentiate BMMs, non-adherent cells were cultured for 3 days with or without Pam3CSK4 (50–100 ng/ml) or lipid A (100 ng/ml) in the presence of macrophage colony-stimulating factor (M-CSF; 20 ng/ml). For the control, phosphate-buffered saline (PBS) was administered instead of the TLR ligands. In some experiments, BMMs were used as osteoclast precursors and further cultured with RANKL (50 ng/ml) in the presence or absence of TLR ligands for 3 days. To identify OCs, cells were fixed with 3.7% formaldehyde and stained with tartrate-resistant acid phosphatase (TRAP) using the Acid Phosphatase Leukocyte Kit (Sigma-Aldrich, St. Louis, MO, USA), and defined as TRAP-positive multinucleated cells (having more than three nuclei). OCs were counted using a microscope. Each condition was tested in triplicate and all experiments were repeated at least three times.

### Reverse transcription (RT)-polymerase chain reaction (PCR) and real-time PCR

Total RNA was extracted from cells using TRIzol reagent (Life Technologies Corporation, Rockville, MD, USA). First strand cDNA was synthesized using 1 mg total RNA with Super Script II RT according to the manufacturer’s instructions (Invitrogen, Grand Island, NY, USA). To detect mRNA expression of Lox-1 and β-actin, RT-PCR was performed using the following: Lox-1 sense 5′-AGACTGGCTCTGGCATAAAG-3′, antisense 5′-AAGGCCAACATGCTTTACAT-3′; β-actin sense 5′-TGAGAGGGAAATCGTGCGT-3′, antisense 5′-GCTGGAAGGTGGACAGTGAG-3′. PCR was performed under the following conditions: 1 min denaturation at 95 °C, 1 min annealing at 55 °C, and 1 min extension at 72 °C, using 36 cycles. PCR products were subjected to electrophoresis on 2% agarose gel and visualized with ethidium bromide. To examine the effects of MAPK inhibitors on Lox-1 mRNA expression, BMCs in the presence of M-CSF (20 ng/ml) were cultured with or without Pam3CSK4 (100 ng/ml) or Lipid A (100 ng/ml) for 3 days after incubation with TLR2/4 inhibitor (25 μM) or MAPK inhibitors, SP600125 (5 μM) as c-Jun N-terminal kinase (JNK) inhibitor or U0126 (10 μM) as MAP/extracellular signal-regulated kinase (ERK) kinase (MEK) inhibitor, respectively, for 1 h. Signals of Lox-1 mRNA were normalized to β-actin mRNA expression levels using Image J software (NIH, Bethesda, MD, USA).

To quantify mRNA levels, cDNA samples were analyzed by quantitative real-time PCR. A total of 1 mg of cDNA was amplified in a 20 μl volume of Power SYBR Green PCR Master Mix (Applied Biosystems, Foster City, CA, USA) in a real-time PCR system (Bio-rad CFX96, Bio-rad Technologies, Inc., Santa Clara, CA, USA) and the fluorescence was monitored at each cycle. Cycle parameters were 95 °C for 30 s to activate Taq followed by 40 cycles of 95 °C for 5 s, 60 °C for 10 s, and 72 °C for 40 s. For real-time analysis, two standard curves were constructed from amplicons for both the β-actin and target gene. Target gene cDNA units in each sample were normalized to β-actin cDNA units. Finally, the relative target gene expression units were expressed as arbitrary units, calculated according to the following formula: relative target gene expression units = target gene cDNA units/β-actin cDNA units. To detect mRNA expression of Lox-1, MyD88 and glyceraldehyde 3-phosphate dehydrogenase (GAPDH), real-time PCR was performed using the following sets: Lox-1 sense 5′-CTGCGAATGACGAGCCTGA-3′, antisense 5′-TCACTGACAACACCAGGCAGAG-3′; MyD88 sense 5′-TACAGGTGGCCAGAGTGGAA-3′, antisense 5′-GCAGTAGCAGATAAAGGCATCGAA-3′; GAPDH sense 5′-TGTGTCCGTCGTGGATCTGA-3′, antisense 5′-TTGCTGTTGAAGTCGCAGGAG′.

### Immunocytochemistry

Cells were fixed in 4% formaldehyde for 5 min and permeabilized with 0.05% Triton-× 100 in phosphate-buffered saline (PBS) for 5 min. Cells were incubated with goat polyclonal anti-rabbit Lox-1 antibody (1:100 dilution, ab60178, Abcam) overnight at 4 °C after blockade of nonspecific binding with 3% goat serum for 40 min at room temperature. Cells treated with primary antibody were washed with PBS and incubated with Alexa fluor 488-conjugated streptavidin (2 μg/ml, Molecular Probes, Eugene, OR) in goat anti-rabbit IgG secondary antibody (1:1500 dilution, Vector Laboratories, Burlingame, CA) for 30 min at room temperature. To label cell nuclei, cells were rinsed in PBS and covered with encapsulant containing 4′,6-diamidino-2-phenylindole (DAPI). Fluorescence was observed using fluorescence microscopy (TMD 300, Nikon).

### FACS analysis

BMCs were cultured for 3 days in α-MEM containing 10% FBS and M-CSF with or without Pam3CSK4 (100 ng/ml) or Lipid A (100 ng/ml) for 3 days. Cells were washed twice with PBS and incubated with anti-Lox-1 and anti-RANK antibodies conjugated with phycoerythrin. The labeled cells were analyzed on a FACS using On Chip (On Chip Bio. Tec., Tokyo, Japan). The ratio of Lox-1- or RANK-positive cells among the total non-adherent BMCs was quantified using the labeled square.

### Western blot analysis

Cells were lysed in Tris-NaCl-Tween (TNT) buffer containing 20 mM tris–HCl (pH 7.5), 200 mM NaCl, 1% Triton X-100, 1 mM dithiothreitol (DTT), and protease inhibitors (Roche, Basel, Switzerland). The protein content of the samples was measured using Pierce reagents, following the manufacturer’s protocol. Protein samples (20 μg) were subjected to sodium dodecyl sulfate-polyacrylamide gel electrophoresis, and proteins were then transferred to a polyvinylidene difluoride (PVDF) membrane (100 V, 1 h, 4 °C). The membranes were incubated with anti-FDPS and anti-β-actin antibodies diluted at 1:1000 in 5% (*w*/*v*) skimmed milk solution supplemented with 0.01% (w/v) azide overnight at 4 °C. The blots were washed in Tris- buffered saline with Tween (TTBS) (10 mM tris–HCl, 50 mM NaCl, 0.25% Tween 20) and incubated with an appropriate secondary antibody for 30 min at room temperature. The immunoreactive proteins were visualized using enhanced chemiluminescence reagents (GE Healthcare, Tokyo, Japan).

### Chemicals

Recombinant human RANKL and M-CSF were purchased from Peprotec Co. Ltd. (Minneapolis, MN, USA). All other chemicals were obtained from Sigma-Aldrich.

### Data analysis

All data are expressed as mean ± standard error of the mean (SEM) of the number of cells (n). Statistical comparisons were performed using analysis of variance (ANOVA). A probability (*P*) of < 0.05 was considered significant.

## Results

### Expression of Lox-1 was upregulated by TLR 2/4 ligands in BMCs

**Fig. 1:**
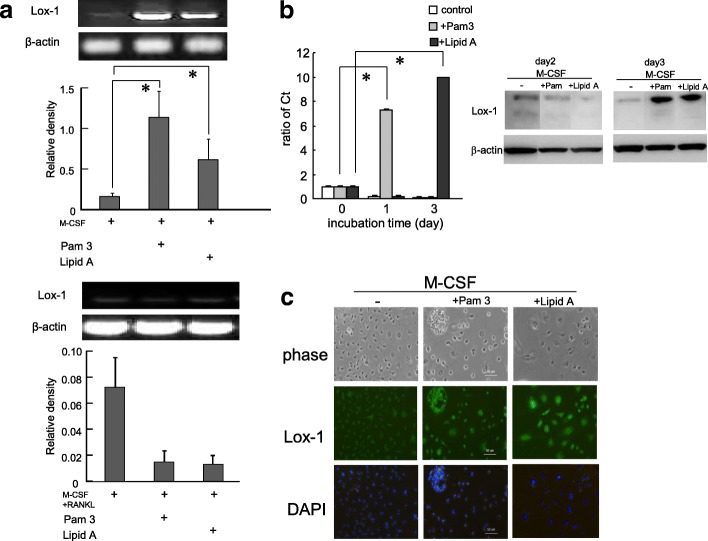
Analysis of LOX-1 in bone marrow cells (BMCs) and bone marrow macrophages (BMMs). (A, upper panel, B, C) BMCs were differentiated into BMMs with or without Pam3CSK4 (TLR2 ligand, 100 ng/ml) or Lipid A (TLR4 ligand, 100 ng/ml) in the presence of macrophage colony-stimulating factor (M-CSF, 20 ng/ml) for 3 days. (A, lower panel) BMMs were cultured with or without Pam3CSK4 (100 ng/ml) or Lipid A (100 ng/ml) in the presence of M-CSF and receptor activator of nuclear factor kappa-B ligand (RANKL, 50 ng/ml) for 3 days. **a** Polymerase chain reaction (PCR) products of LOX-1, a receptor of oxidative low density lipoprotein (oxLDL), mRNA in BMCs (upper panel) and BMMs (lower panel) were amplified using RT-PCR methods. Following stimulation with Pam3CSK4 or Lipid A, Lox-1 expression was upregulated in BMCs (upper panel) but not in BMMs (lower panel). **b** Lox-1 mRNA expression peaked on day 1 or 3 as determined by real-time PCR (left panel) and protein expression on day 3 (right panel). **c** BMCs stimulated with TLR ligands on day 3 were stained with anti-Lox-1 antibody (1:100 dilution) then incubated with Alexa fluor 488-conjugated streptavidin (2 μg/ml) in goat anti-rabbit IgG secondary antibody (1:1500 dilution). Lox-1 expression was upregulated. Data are expressed as mean ± SEM (*n* = 3). *P < 0.05 compared with control

To clarify the effect of TLR ligands on the expression of Lox-1 in BMCs or BMMs, cells were cultured with or without Pam3CSK4 (100 ng/ml) or Lipid A (100 ng/ml) in the presence of M-CSF for 3 days (Fig. 1a). Exposure to Pam3CSK4 or Lipid A significantly upregulated the expression of Lox-1 in BMCs (*p* < 0.05) (upper panel) but suppressed Lox-1 expression in BMMs (lower panel) compared with the control. Lox-1 mRNA expression in BMCs peaked on days 1 or 3 after incubation with Pam3CSK4 or Lipid A as assessed by real-time PCR (*p* < 0.05) (Fig. 1b, left panel). Furthermore, Lox-1 protein expression peaked on day 3 after treatment with both TLR ligands (Fig. 1b, right panel). Similar to the PCR results, protein expression of Lox-1 was also upregulated following exposure to Pam3CSK4 or Lipid A in BMCs as assessed by immunocytochemistry (Fig. 1c).

### TLR2/4 ligands upregulate Lox-1 and RANK in BMCs

**Fig. 2:**
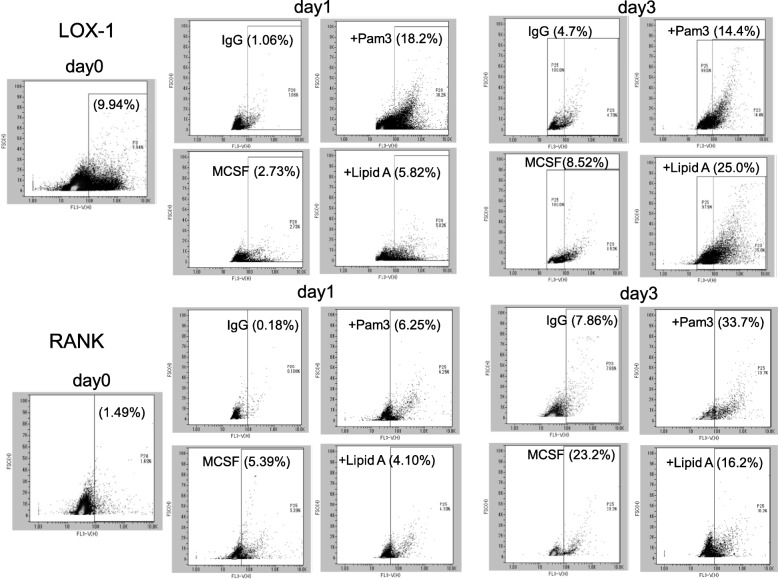
BMC surface expression of Lox-1 and RANK proteins. BMCs were cultured with or without Pam3CSK4 (TLR2 ligand, 100 ng/ml) or Lipid A (TLR4 ligand, 100 ng/ml) in the presence of M-CSF (20 ng/ml) for 3 days. Cells were washed with PBS and incubated with anti-Lox-1 or anti-RANK antibodies. Data are expressed as the mean ± SEM (*n* = 3 experiments)

BMC surface expression of Lox-1 and RANK proteins was analyzed by FACS. Treatment with M-CSF slightly increased the cell surface expression of Lox-1 in BMCs in a time-dependent manner compared with untreated cells (day 0; Fig. 2, upper panel). The addition of Pam3CSK4 or Lipid A significantly upregulated the expression of Lox-1 compared with the control cells (incubated with IgG). Similar to the real-time PCR time course results, Lox-1 expression peaked at day 1 or day 3 after stimulation with Pam3CSK4 or Lipid A, respectively. In the present experiments, RANKL induced the expression of RANK, and the addition of Pam3CSK4 or Lipid A upregulated the expression of RANK in BMCs in a time-dependent manner.

### TLR 2/4 ligands upregulate the expression of Lox-1 in BMCs through activation of MAPK

**Fig. 3:**
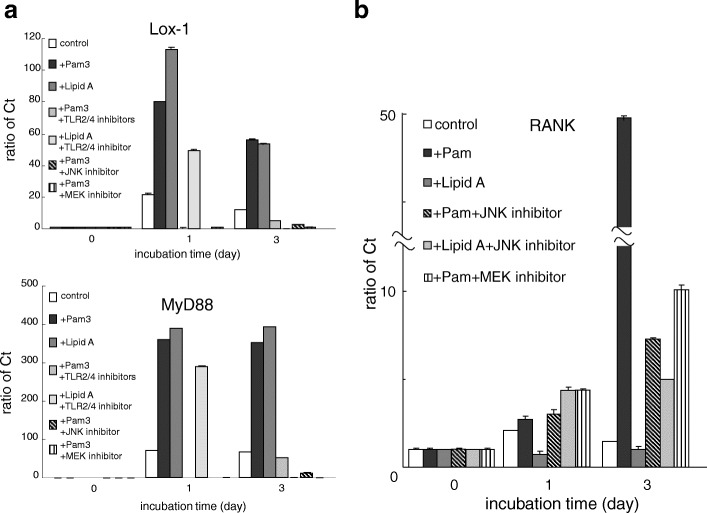
Real time PCR to determine Lox-1, MyD88 and RANK expression in BMCs following addition of inhibitors. BMCs in the presence of M-CSF (20 ng/ml) were cultured with or without Pam3CSK4 (TLR2 ligand, 100 ng/ml) or Lipid A (TLR4 ligand, 100 ng/ml) for 3 days after incubation with TLR2/4 inhibitor (25 μM) or MAPK inhibitors, SP600125 (5 μM) as JNK inhibitor or U0126 (10 μM) as MEK inhibitor, respectively, for 1 h. **a** Both TLR2/4 inhibitor and MAPK inhibitors significantly suppressed TLR 2 or 4 ligand-induced upregulation of Lox-1 (upper panel) and MyD88 (lower panel) mRNA. **b** SP600125 significantly suppressed TLR 2, but not TLR 4 ligand-induced upregulation of RANK mRNA, and U0126 upregulated RANK mRNA. Data are expressed as the mean ± SEM (*n* = 3 experiments)

To assess TLR2 or 4 activation of Lox-1 expression via the downstream pathways of TLR2/4, MyD88, and MAPKs, we examined the effects of these inhibitors on the expression of Lox-1 and RANK in BMCs using real-time PCR. Although the addition of Pam3CSK4 or Lipid A significantly increased the expression of Lox-1 and MyD88 mRNAs, TLR2 or 4 ligand-induced upregulation was reduced in the presence of TLR2/4 and MAPK inhibitor peptides, respectively (Fig. 3a), suggesting an influence towards Lox-1 expression by activation of TLR-MyD88 downstream signaling. Furthermore, the inhibitors of JNK and MEK significantly inhibited the upregulation of TLR2-induced RANK mRNAs on BMCs (Fig. 3b). These results demonstrated that TLR2/4 ligands activated Lox-1 expression through the MEK and JNK activation pathway, including MyD88.

### TLRs stimulation with oxLDL accelerates osteoclastogenesis at early-phase

**Fig. 4:**
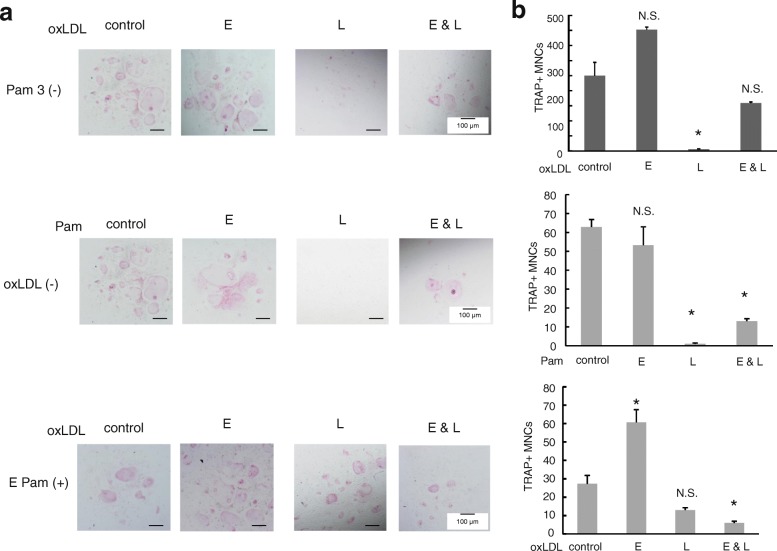
Effect of stimulation to TLRs and/or Lox-1 to TRAP-positive multinucleated cells. The effect of stimulation to Lox-1 with oxLDL and/or to TLR 2 with Pam3CSK4 was examined using TRAP staining. In each panel, E indicates stimulation at early-phase (BMC: day 0–3 before stimulation with RANKL) and L indicates stimulation at late-phase (BMMs: day 4–6 after stimulation with RANKL). (**a** and **b** upper) BMCs in the presence of M-CSF or BMMs in the presence of M-CSF and RANKL were cultured with oxLDL (30 μg/ml) at indicated phase. (**a** and **b** middle) BMCs in the presence of M-CSF or BMMs in the presence of M-CSF and RANKL were cultured with Pam3CSK4 (10 ng/ml) at indicated phase. (**a** and **b** lower) BMCs in the presence of M-CSF and Pam3CSK4 (25 ng/ml) were stimulated with oxLDL (15 μg/ml) at early- or late-phase. Data are expressed as the mean ± SEM (*n* = 3 experiments). *P < 0.05 compared with control

To clarify TLR2-induced Lox-1 upregulation on osteoclastogenesis, we examined the effect of oxLDL (30 μg/ml) on osteoclast differentiation on early-phase (BMC: day 0–3 before stimulation with RANKL) and/or on late-phase (BMM: day 0–3 after RANKL stimulation) osteoclastogenesis. The number of TRAP-positive multinucleated cells were increased at early phase and decreased at late phase, by addition of only oxLDL (Fig. 4a and b each upper panels). Furthermore, Pam3CSK4 (10 ng/ml) had no effect on early-phase, but an inhibition effect at late-phase osteoclastogenesis (Fig. 4a and b each middle panels). When we stimulated BMCs with both Pam3CSK4 (25 ng/ml) and oxLDL (15 μg/ml) at early-phase, numbers of TRAP-positive multinucleated cells were significantly increased (Fig. 4a and b each lower panels).

## Discussion

P. gingivalis is known to stimulate TLR2 and/or TLR4 [11, 12] via MyD88 to modulate osteoclastogenesis [18, 40–42], resulting in alveolar bone resorption through differential regulation of TLR2 and TLR4 signaling pathways in a RANKL-dependent manner [13]. Although Lipid A, a TLR4 ligand, has been shown to be a potent stimulator of bone loss in vivo [14], little is known about whether other TLR ligands have a stimulatory or inhibitory effect on osteoclastogenesis in vitro. Furthermore, many clinical studies show that osteoporosis is associated with atherosclerosis [43, 44]. Although atherosclerosis is reported to result in inhibited bone formation because oxLDL blocked differentiation of osteoblast progenitor cells [45, 46], little is known about how dyslipidemia influences bone resorption. In the present study, we found that TLRs upregulated the oxLDL receptor, Lox-1, via MAPK in BMCs, resulting in promotion of osteoclastogenesis. This finding suggests that periodontitis would worsen with progression of dyslipidemia.

The subcutaneous or intraperitoneal administration of Pam3CSK4 is reported to upregulate bone resorption in vivo, suggesting that Pam3CSK4 directly promotes osteoclastogenesis from BMMs after RANKL priming [41, 47]. However, synthetic TLR2 ligand inhibits OC formation in BMMs treated with M-CSF and RANKL in vitro [18, 19] Furthermore, combination of RANKL and LPS inhibited osteoclastogenesis [19, 20]. Our data also showed that numbers of TRAP-positive cells were decreased when BMMs were stimulated with Pam3CSK, compared with those treated with M-CSF and RANKL. In contrast, the addition of Pam3CSK and oxLDL, a Lox-1 ligand together at early phase, increased the number of TRAP-positive cells. Although the mechanism through which P. gingivalis-induces bone resorption via TLR2 and TLR4 is not fully understood, our results suggest that TLR-stimulation in periodontitis upregulates alveolar bone resorption under high plasma concentration of oxLDL in dyslipidemia.

OC progenitors, including BMCs and BMMs, are reported to express abundant levels of LDL receptors, such as Lox-1, in a RANKL-independent manner. Expression levels of Lox-1 are greatly decreased during osteoclastogenesis, particularly during the fusion of mononuclear cells to form multinuclear OCs [48]. The present data showed that Lox-1 expression was upregulated by TLR 2 or 4 ligands in BMCs but not in BMMs. Periodontal tissues in ApoE^−/−^ hyperlipidemia model rats are reported to exhibit more TRAP-positive multinuclear cells and increased TLR2 and TLR4 expression levels [33], suggesting that high oxLDL concentration effects osteoclastogenesis via the elevation of TLRs. OxLDL is reported to suppress RANKL-induced osteoclastogenesis, TRAP activity, and bone-resorbing activity derived from human peripheral blood mononuclear cells in vitro. This suppression is suggested to be caused by RANKL-induced phosphorylation of ERK, p38, and JNK kinases, together with the suppression of DNA binding activities of transcriptional factors, such as nuclear factor-kappa B (NF-kappa-B) and nuclear fsctor of activated T-cells (NFAT) [49]. Our data demonstrated that TLR 2 or 4 ligand-induced Lox-1 upregulation was reduced in the presence of TLR2/4 and MAPK inhibitor, respectively, and the inhibitors of JNK and MEK significantly inhibited the upregulation of TLR 2-induced RANK mRNA on BMCs. We also found that TLR-stimulation together with oxLDL accelerated early-phase but not late-phase osteoclastogenesis, and only oxLDL stimulation suppressed RANKL-induced osteoclastogenesis, consistent with previous studies. In the present study, Pam3CSK4 was shown to upregulate the expression of RANK and Lox-1 in BMCs. Furthermore, stimulation with both Pam3CSK4 and oxLDL was shown to promote progression of osteoclastogenesis, suggesting that oxLDL-Lox-1 signaling promotes osteoclastogenesis through expression of Lox-1 by stimulating TLRs.

Similar reports have shown that plasma lipids likely play a role in maintaining bone mass [48, 50]. A better understanding of the coupling between osteoporosis and atherosclerosis is indicated in previous studies whereby treatment of one disease may have beneficial effects on another [51–56]. The American Heart Association reported in 2012 that although periodontal interventions result in a reduction in systemic inflammation and endothelial dysfunction in short-term studies, there is no evidence that they prevent atherosclerotic cardiovascular disease (ASVD) or modify its outcomes [57]. Although our data could not support that ASVD developed due to periodontitis worsening dyslipidemia, it did show that osteoclastogenesis was upregulated by TLR-stimulation, like bacterial infection, in periodontitis with dyslipidemia.

In this study, we focused on elucidating the correlation between dyslipidemia and progression of periodontitis. Our results indicated that stimulation of BMCs with TLRs, representative of a bacterial infection, upregulated Lox-1 expression resulting in promotion of osteoclastogenesis in the presence of oxLDL in dyslipidemia. Although the present data did not clarify the direct relationships between periodontitis and atherosclerotic vascular disease, periodontitis at least partially worsens by the complication of dyslipidemia.

## Conclusion

In conclusion, the activation of TLR 2 and/or 4 upregulated the expression of Lox-1 through activation of MAPK in BMCs but not in osteoblasts, suggesting the promotion of osteoclastogenesis during dyslipidemia.

## Abbreviations

ApoE: Apolipoprotein E
ASVD: Atherosclerotic cardiovascular disease
BMC: Bone marrow cell
BMM: Bone marrow macrophage
ERK: Extracellular signal-regulated kinase
FACS: Fluorescence activated cell sorting
JNK: c-Jun N-terminal kinase
Lox-1: Lectin-like oxidized low-density-lipoprotein receptor 1
LPS: Lipopolysaccharide
MAPK: Mitogen-activated protein kinase
M-CSF: Macrophage colony-stimulating factor
MEK: MAPK/ERK kinase
MyD88: Myeloid differentiation factor 88
NFAT: Nuclear factor of activated T-cells
NF-kappa-b: Nuclear factor-kappa B
OC: Osteoclast
oxLDL: oxidized low-density lipoprotein
P. gingivalis: *Porphyromonas gingivalis*
PCR: Polymerase chain reaction
RANKL: Receptor activator of nuclear factor kappa-B ligand
TLR: Toll-like receptor
TRAP: Tartrate-resistant acid phosphatase
